# Clustering of reads with alignment-free measures and quality values

**DOI:** 10.1186/s13015-014-0029-x

**Published:** 2015-01-28

**Authors:** Matteo Comin, Andrea Leoni, Michele Schimd

**Affiliations:** Department of Information Engineering, University of Padova, Padova, Italy

**Keywords:** Alignment-free measures, Reads quality values, Reads clustering

## Abstract

**Background:**

The data volume generated by Next-Generation Sequencing (NGS) technologies is growing at a pace that is now challenging the storage and data processing capacities of modern computer systems. In this context an important aspect is the reduction of data complexity by collapsing redundant reads in a single cluster to improve the run time, memory requirements, and quality of post-processing steps like assembly and error correction. Several alignment-free measures, based on *k*-mers counts, have been used to cluster reads.

Quality scores produced by NGS platforms are fundamental for various analysis of NGS data like reads mapping and error detection. Moreover future-generation sequencing platforms will produce long reads but with a large number of erroneous bases (up to 15 *%*).

**Results:**

In this scenario it will be fundamental to exploit quality value information within the alignment-free framework. To the best of our knowledge this is the first study that incorporates quality value information and *k*-mers counts, in the context of alignment-free measures, for the comparison of reads data. Based on this principles, in this paper we present a family of alignment-free measures called *D*^*q*^-type. A set of experiments on simulated and real reads data confirms that the new measures are superior to other classical alignment-free statistics, especially when erroneous reads are considered. Also results on *de novo* assembly and metagenomic reads classification show that the introduction of quality values improves over standard alignment-free measures. These statistics are implemented in a software called QCluster (http://www.dei.unipd.it/~ciompin/main/qcluster.html).

## Background

The data volume generated by Next-Generation Sequencing (NGS) technologies is growing at a pace that is now challenging the storage and data processing capacities of modern computer systems [1]. Current technologies produce over 500 billion bases of DNA per run, and the forthcoming sequencers promise to increase this throughput. The rapid improvement of sequencing technologies has enabled a number of different sequencing-based applications like genome resequencing, RNA-Seq, ChIP-Seq and many others [2]. Handling and processing such large files is becoming one of the major challenges in most genome research projects.

Alignment-based methods have been used for quite some time to establish similarity between sequences [3]. However there are cases where alignment methods can not be applied or they are not suited.

For example the comparison of whole genomes is impossible to conduct with traditional alignment techniques, because of events like rearrangements that can not be captured with an alignment [4-6]. Although fast alignment heuristics exist, another drawback is that alignment methods are usually time consuming, thus they are not suited for large-scale sequence data produced by Next-Generation Sequencing technologies (NGS) [7,8]. For these reasons a number of alignment-free techniques have been proposed over the years [9].

The use of alignment-free methods for comparing sequences has proved useful in different applications. Researchers have shown that the use of *k*-mers frequencies can improve the construction of phylogenetic trees traditionally based on a multiple-sequence alignment, especially for distant related species [10]. Some alignment-free measures use the patterns distribution to study evolutionary relationships among different organisms [4,11,12]. The efficiency of alignment-free measures also allows the reconstruction of phylogenies for whole genomes [4-6]. Several alignment-free methods have been devised for the detection of enhancers in ChIP-Seq data [13-15] and also of entropic profiles [16,17]. Another application is the classification of protein remotely related, which can be addressed with sophisticated word counting procedures [18,19]. The assembly-free comparison of genomes based on NGS reads has been investigated only recently [7,8]. For a comprehensive review of alignment-free measures and applications we refer the reader to [9].

In this study we want to explore the ability of alignment-free measures to cluster reads data. Clustering techniques are widely used in many different applications based on NGS data, from error correction [20] to the discovery of groups of microRNAs [21]. With the increasing throughput of NGS technologies another important aspect is the reduction of data complexity by collapsing redundant reads into a single cluster to improve the run time, memory requirements, and quality of subsequent steps like assembly.

In [22] Solovyov *et al.* presented one of the first comparison of alignment-free measures when applied to NGS reads clustering. They focused on clustering reads coming from different genes and different species based on *k*-mer counts. They showed that *D*-type measures (see next section), in particular $D_{2}^{*}$, can efficiently detect and cluster reads from the same gene or species (as opposed to [21] where the clustering is focused on errors). In this paper we extend this study by incorporating quality value information into these measures.

Quality scores produced by NGS platforms are fundamental for various analysis of NGS data: mapping reads to a reference genome [23]; error correction [20]; detection of insertion and deletion [24] and many others. Moreover future-generation sequencing technologies will produce longer and less biased reads with a large number of erroneous bases [25]. The average number of errors per read will grow up to 15 *%*, thus it will be fundamental to exploit quality value information within the alignment-free framework and the *de novo* assembly where longer and less biased reads could have dramatic impact.

Most applications require as input a set of reads that is error-free, thus they need to pre-process the data with a filter. Usually quality values are used to detect low quality reads, that in most applications are discarded. With the increasing of error rates, the ability to work with erroneous reads will be fundamental. Moreover, in this scenario, quality values are used only during the pre-process to select reads that are error-free. Approximately half of the data produced by a sequencers are quality values, yet they are discarded after the pre-processing. In this paper we pave the way to a new paradigm where also quality values play a major role when analyzing reads data.

In the following section we briefly review some alignment-free measures. Then we present a new family of statistics, called *D*^*q*^-type^a^, that take advantage of quality values. The software QCluster is discussed and relevant results on simulated and real data are presented in the results section. In the last section we summarize the findings and we discuss future directions of investigation.

## Previous work on alignment-free measures

One of the first papers that introduced an alignment-free method is due to Blaisdell in 1986 [26]. He proposed a statistic called *D*_2_, to study the correlation between two sequences. The initial purpose was to speed up database searches, where alignment-based methods were too slow. The *D*_2_ similarity is the correlation between the number of occurrences of all *k*-mers appearing in two sequences. Let *X* and *Y* be two sequences from an alphabet *Σ*. The value *X*_*w*_ is the number of times *w* appears in *X*, with possible overlaps. Then the *D*_2_ statistic is:

$$ D_{2}= \sum_{w \in \Sigma^{k}} X_{w} Y_{w}. $$

This is the inner product of the word vectors *X*_*w*_ and *Y*_*w*_, each one representing the number of occurrences of words of length *k*, *i.e.k*-mers, in the two sequences. However, it was shown by Lippert *et al.* [27] that the *D*_2_ statistic can be biased by the stochastic noise in each sequence. To address this issue another popular statistic, called ${D_{2}^{z}}$, was introduced in [14]. This measure was proposed to standardize the *D*_2_ in the following manner:

$${D_{2}^{z}} = \frac{D_{2} - \mathbb{E}(D_{2})} {\mathbb{V}(D_{2})}, $$

where $\mathbb {E}(D_{2})$ and $\mathbb {V}(D_{2})$ are the expectation and the standard deviation of *D*_2_, respectively. Although the ${D_{2}^{z}}$ similarity improves *D*_2_, it is still dominated by the specific variation of each pattern from the background [28,29]. To account for different distributions of the *k*-mers, in [28,29] two other new statistics are defined and named $D_{2}^{*}$ and ${D_{2}^{s}}$. Let $\tilde {X}_{w}=X_{w} - (n-k+1)*p_{w}$ and $\tilde {Y}_{w}=Y_{w} - (n-k+1)*p_{w}$ where *p*_*w*_ is the probability of *w* under the null model. Then $D_{2}^{*}$ and ${D_{2}^{s}}$ can be defined as follows:

$$D_{2}^{*} = \sum _{w \in \Sigma^{k}} \frac{\tilde{X}_{w} \tilde{Y}_{w}}{(n-k+1)p_{w}} $$

and,

$$D_{2}^{s} = \sum_{w \in \Sigma^{k}} \frac{\tilde{X}_{w} \tilde{Y}_{w}}{\sqrt{\tilde{X}^{2}_{w} + \tilde{Y}^{2}_{w}}}. $$

This latter similarity measure responds to the need of normalization of *D*_2_. These set of alignment-free measures are usually called *D*-type statistics. All these statistics have been studied by Reinert *et al.* [28] and Wan *et al.* [29] for the detection of regulatory sequences. From the word vectors *X*_*w*_ and *Y*_*w*_ several other measures can be computed like *L*_2_, Kullback-Leibler divergence (KL), symmetrized KL [22] etc.

## Comparison of reads with quality values

### Background on quality values

Upon producing base calls for a read *x*, sequencing machines also assign a *quality scoreQ*_*x*_(*i*) to each base in the read. These scores are usually given as *phred*-scaled probability [30] of the *i*-th base being wrong

$${} Q_{x}(i) = -10 \log_{10}{Prob\{\text{the base \textit{i} of read \textit{x} is wrong }\}}. $$

For example, if *Q*_*x*_(*i*)=30 then there is 1 in 1000 chance that base *i* of read *x* is incorrect.

If we assume that quality values are produced independently to each other (similarly to [23]), we can calculate the probability of an entire read *x* being correct as:

$$P_{x}\left\{\text{the read \textit{x} is correct}\right\}= \prod_{j=0}^{n-1}{\left(1- 10^{- Q_{x}(j)/{10}}\right)} $$

where *n* is the length of the read *x*. In the same way we define the probability of a word *w* of length *k*, occurring at position *i* of read *x* being correct as:

$$\begin{aligned} P_{w,i}&\left\{\text{the word \textit{w} at position \textit{i} of read \textit{x} is correct}\right\}\\ &= \prod_{j=0}^{k-1}{\left(1- 10^{-Q_{x}(i+j)/{10}}\right)}. \end{aligned} $$

In all previous alignment-free statistics the *k*-mers are counted such that each occurrence contributed as 1 irrespective of its quality. Here we can use the quality of that occurrence instead to account also for erroneous *k*-mers. The idea is to model sequencing as the process of reading *k*-mers from the reference and assigning a probability to them. Thus this formula can be used to weight the occurrences of all *k*-mers used in the previous statistics.

### New *D*^*q*^-type statistics

We extend here *D*-type statistics [28,29] to account for quality values. By defining ${X_{w}^{q}}$ as the sum of probabilities of all the occurrences of *w* in *x*:

$$X_{w}^{q} = \sum_{i \in \left\{i| \text{\textit{w} occurs in \textit{x} at position \textit{i}}\right\} }P_{w,i} $$

we assign a weight (*i.e.* a probability) to each occurrence of *w*. Now ${X_{w}^{q}}$ can be used instead of *X*_*w*_ to compute the alignment-free statistics. Note that, by using ${X_{w}^{q}}$, every occurrence is not counted as 1, but with a value in [ 0,1] depending of the reliability of the read. We can now define a new alignment-free statistic as :

$$ {D_{2}^{q}}= \sum_{w \in \Sigma^{k}} {X_{w}^{q}} {Y_{w}^{q}}. $$

This is the extension of the *D*_2_ measure, in which occurrences are weighted based on quality scores. Following the previous section we can also define the centralized *k*-mers counts as follows:

$$ \tilde{{X_{w}^{q}}} = {X_{w}^{q}} - (n-k+1)p_{w} E(P_{w}) $$

where *n*=|*x*| is the length of *x*, *p*_*w*_ is the probability of the word *w* in the i.i.d. model and the expected number of occurrences (*n*−*k*+1)*p*_*w*_ is multiplied by *E*(*P*_*w*_) which represents the expected probability of *k*-mer *w* based on the quality scores.

We can now extend two other popular alignment-free statistics:

$$D_{2}^{*q} = \sum_{w \in \Sigma^{k}} \frac{\tilde{{X_{w}^{q}}} \tilde{{Y_{w}^{q}}}}{(n-k+1)p_{w} E(P_{w})} $$

and,

$$D_{2}^{sq} = \sum_{w \in \Sigma^{k}} \frac{\tilde{{X_{w}^{q}}}\tilde{{Y_{w}^{q}}}}{\sqrt{\tilde{{X_{w}^{q}}}^{2} + \tilde{{Y_{w}^{q}}}^{2}}}. $$

We call these three alignment-free measures *D*^*q*^-type. Now, *E*(*P*_*w*_) depends on *w* and on the actual sequencing machine, therefore it can be very hard, if not impossible, to calculate precisely. However, if the set  of all the reads is large enough we can estimate the prior probability using the posterior relative frequency, *i.e.* the frequency observed on the actual set , similarly to [23]. We assume that, given the quality values, the error probability on a base is independent from its position within the read and from all other quality values (see [23]). We defined two different approximations, the first one estimates *E*(*P*_*w*_) as the average error probability of the *k*-mer *w* among all reads $x \in \mathbb {D}$:

$$E(P_{w})\approx \frac{\sum_{x \in \mathbb{D}} {X_{w}^{q}} }{\sum_{x \in \mathbb{D}} X_{w}} $$

while the second defines, for each base *j* of *w*, the average quality observed over all occurrences of *w* in :

$$\overline{Q_{w}}[\!j]=\frac{\sum_{x \in \mathbb{D}} \sum_{i \in \{i| \text{ \textit{w} occurs in \textit{x} at position \textit{i}} \} } Q_{x}(i+j)}{\sum_{x \in \mathbb{D}} X_{w}} $$

and it uses the average quality values to compute the expected word probability.

$$E(P_{w})\approx \prod_{j=0}^{k-1}{\left(1- 10^{-\overline{Q_{w}}(j)/{10}}\right)} $$

We called the first approximation *Average Word Probability (AWP)* and the second one *Average Quality Probability (AQP)*. Both these approximations are implemented within the software QCluster and tests are presented in the Experimental Results section.

### Quality value redistribution

If we consider the meaning of quality values it is possible to further exploit it to extend and improve the above statistics. Let’s say that the base *A* has quality 70%, it means that there is a 70% probability that the base is correct. However there is also another 30% probability that the base is incorrect. Let’s ignore for the moment insertion and deletion errors, if the four bases are equiprobable, this means that with uniform probability 10% the wrong base is a *C*, or a *G* or a *T*. It’s therefore possible to redistribute the “missing quality” among other bases.

We can perform a more precise operation by redistributing the missing quality among other bases in proportion to their frequency in the read. For example, if the frequencies of the bases in the read are *A* =20%, *C* =30%, *G* =30%, *T* =20%, the resulting qualities, after the redistribution, will be: *A* =70%, *C*=30*%*∗30*%*/(30*%*+30*%*+20*%*)=11.25*%*, *G*=30*%*∗30*%*/(30*%*+30*%*+20*%*)=11.25*%*, *T*=30*%*∗20*%*/(30*%*+30*%*+20*%*)=7.5*%*. For an example see Table 1.

**Table 1:** **Example of quality value redistribution of the word** ***T*** ***G*** ***A*** ***C*** ***C*** ***A***

Original Word	T	G	A	C	C	A
Accuracy	X	X	70%	X	X	X
Possible Word 1	T	G	C	C	C	A
Accuracy	X	X	11.25%	X	X	X
Possible Word 2	T	G	G	C	C	A
Accuracy	X	X	11.25%	X	X	X
Possible Word 3	T	G	T	C	C	A
Accuracy	X	X	7.5%	X	X	X

The same redistribution, with a slight approximation, can be extended to *k*-mers quality. More in detail, we consider the case in which only one base is wrong, thus we redistribute the quality of only one base at a time. Given a *k*-mer, we generate all neighboring words that can be obtained by substitution of the wrong base. The quality of the replaced letter is calculated as in the previous example and the quality of the entire word is again given by the product of the qualities of all the bases in the new *k*-mers. We increment the corresponding entry of the vector ${X_{w}^{q}}$ with the score obtained for the new *k*-mer. This process is repeated for all bases of the original *k*-mer. Thus every time we are evaluating the quality of a word, we are also scoring neighboring *k*-mers by redistributing the qualities. We didn’t consider the case where two or more bases are wrong simultaneously, because the computational cost would be too high and the quality of the resulting word would not appreciably affect the measures.

## QCluster: clustering of reads with *D*^*q*^-type measures

Clustering is the process of partitioning a given set into *c* distinct disjoint subsets called *clusters* such that elements (*e.g.* reads) on the same cluster have minimum distance between them and maximum distance with elements of different clusters. Centroid clustering associates to each cluster one point on the space of input elements called *centroid* which does not need to be part of the input set. Each element is then assigned to the cluster for which the distance measure to the centroid is minimized. A classical example of centroid clustering is the algorithm k-means.

We extent the software afcluster [22] which uses k-means to compute the clustering of reads based on several distance measures: *L*_2_ which is the Euclidean norm, Kullback-Liebler divergence and its symmetrized version, and *D*_2_ based measures. Starting from this software we developed QCluster by incorporating the computation of the ${D_{2}^{q}}$-type statistics described above using both *AWP* and *AQP* prior probability estimators and the redistribution of quality values.

The program takes in input a FastQ format file and performs centroid-based clustering (k-means) of the reads based on the counts and the quality of *k*-mers. When using the *D*^*q*^-type measures, one needs to choose the method for the computation of the expected word probability, *AWP* or *AQP*, and the quality redistribution.

Since some of the implemented distances (symmetrized KL, $D^{*}_{2}$) do not guarantee to converge, we implemented a stopping criteria. The execution of the algorithm interrupts if the number of iterations without improvements, over the best solution, exceeds a certain threshold. In this case, the best solution found is returned. To avoid as much as possible biases due to the initial random generation of centroids, the best solution over several runs is reported. The number of runs may be set by the user and for our experiments we use the value 5.

Several other options like consensus clustering, reverse complement and different normalizations are available. All implemented measures can be computed in linear time and space, which is desirable for large NGS datasets. The QCluster is freely available (http://www.dei.unipd.it/~ciompin/main/qcluster.html), it has been implemented in C++ and compiled and tested using GNU GCC.

## Experimental results

Several tests have been performed in order to estimate the effectiveness of the different distances, on both simulated and real datasets. In particular, we had to ensure that, with the use of the additional information of quality values, the clustering improved compared to that produced by the original algorithms.

For simulations we use the dataset of human mRNA genes downloaded from NCBI [31], also used in [22]. We randomly select 50 sets of 100 sequences each of human mRNA, with the length of each sequence ranged between 500 and 10000 bases. From each sequence, 10000 reads of length 200 were simulated using Mason [32,33] with different parameters, *e.g.* percentage of mismatches, read length. We apply QCluster using different distances, to the whole set of reads and then we measure the quality of the clusters produced by evaluating the extent to which the partitioning agrees with the natural splitting of the sequences. In other words, we measured how well reads originating from the same sequence are grouped together. We calculate the recall rate as follows, for each mRNA sequence *S* we identify the set of reads originated from *S*. We look for the cluster *C* that contains most of the reads of *S*. The percentage of the *S* reads that have been grouped in *C* is the recall value for the sequence *S*. We repeat the same operation for each sequence and calculate the average value of recall rate over all sequences.

Several clustering were produced by using the following distance types: $D^{*}_{2}$, *D*_2_, *L*_2_, *KL*, symmetrized *KL* and compared with $D_{2}^{*q}$ in all its variants, using the expectation formula (1) *AWP* or (2) *AQP*, with and without quality redistribution (q-red). In order to avoid as much as possible biases due to the initial random generation of centroids, each algorithm was executed 5 times with different random seeds and the clustering with the lower distortion was chosen.

Table 2 reports the recall while varying error rates, number of clusters and *k*. As expected, for all distances the recall rate decreases with the number of clusters. For traditional distances, if the reads do not contain errors then $D^{*}_{2}$ preforms consistently better then the others *D*_2_, *L*_2_, *KL*. When the sequencing process becomes more noisy, the *KL* distances appears to be less sensitive to sequencing errors. However if quality information are used, $D^{*q}_{2}$ outperforms all other methods and the advantage grows with the error rate. This confirms that the use of quality values can improve clustering accuracy. When the number of clusters increases then the advantage of $D^{*q}_{2}$ becomes more evident. In these experiments the use of *AQP* for expectation within $D^{*q}_{2}$ is more stable and better performing compared with formula *AWP*. The contribution of quality redistribution (q-red) is limited, although it seems to have some positive effect with the expectation *AQP*.

**Table 2:** **Recall rates of clustering of mRNA simulated reads (10000 reads of length 200) for different measures, error rates, number of clusters and parameter** ***k***

	***k*** **=2**					***k*** **=3**			
	**(a)**					**(b)**			
**Distance**	**No errors**	**3** ***%***	**5** ***%***	**10** ***%***		**No errors**	**3** ***%***	**5** ***%***	**10** ***%***
	**2 clusters**					**2 clusters**			
$D^{*}_{2}$	**0,815**	0,813	0,810	0,801		**0,822**	0,819	0,814	0,794
$D^{*q}_{2}$ *AQP*	**0,815**	**0,815**	**0,813**	**0,810**		**0,822**	**0,822**	**0,820**	**0,809**
$D^{*q}_{2}$ *AQP* q-red	**0,815**	**0,815**	**0,813**	**0,810**		**0,822**	**0,822**	**0,820**	0,807
$D^{*q}_{2}$ *AWP*	0,809	0,806	0,805	0,802		0,809	0,807	0,805	0,802
$D^{*q}_{2}$ *AWP* q-red	0,809	0,806	0,805	0,802		0,809	0,807	0,805	0,802
*L*_2_	0,811	0,807	0,806	0,801		0,810	0,806	0,805	0,801
KL	0,812	0,809	0,807	0,802		0,812	0,809	0,807	0,802
Symm, KL	0,812	0,809	0,807	0,802		0,812	0,808	0,806	0,802
*D*_2_	0,811	0,807	0,806	0,801		0,809	0,806	0,805	0,800
	**3 clusters**					**3 clusters**			
$D^{*}_{2}$	**0,695**	0,689	0,683	0,662		**0,717**	0,707	0,697	0,668
$D^{*q}_{2}$ *AQP*	**0,695**	**0,696**	**0,696**	0,689		**0,717**	0,711	**0,705**	0,679
$D^{*q}_{2}$ *AQP* q-red	**0,695**	**0,696**	**0,696**	**0,691**		**0,717**	**0,712**	0,704	**0,681**
$D^{*q}_{2}$ *AWP*	0,653	0,646	0,646	0,638		0,668	0,662	0,655	0,646
$D^{*q}_{2}$ *AWP* q-red	0,653	0,646	0,645	0,637		0,668	0,662	0,655	0,644
*L*_2_	0,682	0,673	0,671	0,657		0,685	0,677	0,674	0,663
KL	0,694	0,687	0,685	0,672		0,696	0,689	0,687	0,675
Symm, KL	0,693	0,686	0,684	0,669		0,695	0,688	0,685	0,673
*D*_2_	0,675	0,668	0,662	0,654		0,675	0,671	0,665	0,655
	**4 clusters**					**4 clusters**			
$D^{*}_{2}$	**0,623**	0,613	0,606	0,574		0,627	0,616	0,591	0,551
$D^{*q}_{2}$ *AQP*	0,622	0,621	0,618	0,602		**0,628**	**0,617**	0,602	0,572
$D^{*q}_{2}$ *AQP* q-red	0,622	**0,622**	**0,619**	**0,605**		**0,628**	**0,617**	**0,603**	**0,573**
$D^{*q}_{2}$ *AWP*	0,580	0,563	0,566	0,535		0,582	0,571	0,572	0,555
$D^{*q}_{2}$ *AWP* q-red	0,580	0,560	0,565	0,533		0,582	0,570	0,570	0,555
*L*_2_	0,554	0,551	0,547	0,540		0,568	0,565	0,553	0,543
KL	0,555	0,548	0,545	0,536		0,566	0,558	0,547	0,537
Symm, KL	0,556	0,549	0,546	0,538		0,562	0,554	0,547	0,539
*D*_2_	0,553	0,547	0,547	0,538		0,556	0,549	0,548	0,540
	**5 clusters**					**5 clusters**			
$D^{*}_{2}$	0,553	0,539	0,532	0,500		0,560	0,534	0,512	0,462
$D^{*q}_{2}$ *AQP*	**0,554**	**0,545**	**0,551**	0,532		0,560	0,544	0,524	**0,489**
$D^{*q}_{2}$ *AQP* q-red	0,553	0,544	0,550	**0,533**		**0,561**	**0,545**	**0,531**	0,487
$D^{*q}_{2}$ *AWP*	0,483	0,475	0,470	0,463		0,509	0,494	0,485	0,470
$D^{*q}_{2}$ *AWP* q-red	0,483	0,475	0,470	0,461		0,509	0,494	0,482	0,470
*L*_2_	0,478	0,472	0,465	0,453		0,500	0,495	0,486	0,465
KL	0,498	0,488	0,484	0,468		0,507	0,501	0,492	0,476
Symm, KL	0,498	0,488	0,484	0,468		0,507	0,500	0,491	0,474
*D*_2_	0,470	0,464	0,457	0,449		0,488	0,482	0,476	0,455

Best results are in bold.

In a second series of experiments, maintaining the previously described experimental setup, we test how the number of reads and the different types of errors affect the recall rates. Table 3 shows the recall rates, for different methods, while varying the number of reads and the types of sequencing errors. The relative performances are similar to that of Table 2, however we can note that as the number of reads increases the advantage of quality based measures slightly improve. It is of interest to note that among the different types of sequencing errors, deletions seem to cause a drop of recall rates more evident than mismatches and insertions.

**Table 3:** **Recall rates of clustering of mRNA simulated reads (reads of length 200,** ***k*** **=2 and 2 clusters) for different measures, different types of errors and number of reads**

**Distance**	**No errors**	**Mismatch = 10%**	**Insertion = 10%**	**Deletion = 10%**
			**Mismatch = 10%**	**Mismatch = 10%**
**500 reads**				
$D^{*}_{2}$	0.86445887	0.83981814	0.79073482	0.80640363
$D^{*q}_{2}$ *AQP*	0.86441326	**0.86694192**	**0.86376933**	**0.85925575**
$D^{*q}_{2}$ *AQP* q-red	0.86441326	0.86375045	0.85782736	0.85818320
$D^{*q}_{2}$ *AWP*	0.86723257	0.85428665	0.84756397	0.85088665
$D^{*q}_{2}$ *AWP* q-red	0.86723257	0.85613671	0.85305013	0.85504185
*L*_2_	0.86114263	0.85504302	0.85105192	0.85118905
*D*_2_	0.86258900	0.85247832	0.84995366	0.85110380
KL	**0.87235487**	0.85916040	0.85026923	0.85475077
Simm, KL	0.86712365	0.85695963	0.84730941	0.85418699
**1000 reads**				
	0.86594479	0.83906192	0.78782226	0.80686962
$D^{*q}_{2}$ *AQP*	0.86599548	**0.86400152**	**0.86423642**	**0.85659489**
$D^{*q}_{2}$ *AQP* q-red	0.86600096	0.86099042	0.85469494	0.85441545
$D^{*q}_{2}$ *AWP*	0.86790093	0.85433807	0.84230775	0.84839892
$D^{*q}_{2}$ *AWP* q-red	0.86790093	0.85770704	0.85062824	0.85104321
*L*_2_	0.86216987	0.85477261	0.84904670	0.85024936
*D*_2_	0.86058645	0.85312555	0.84767965	0.85043005
KL	**0.87048717**	0.85667036	0.85002398	0.85088847
Simm, KL	0.86919513	0.85488101	0.84896184	0.84950072
**2000 reads**				
$D^{*}_{2}$	0.86307749	0.83460148	0.78680210	0.81273009
$D^{*q}_{2}$ *AQP*	0.86306541	**0.86490821**	**0.86432381**	**0.85783381**
$D^{*q}_{2}$ *AQP* q-red	0.86306541	0.86129411	0.85330127	0.85111236
$D^{*q}_{2}$ *AWP*	0.86305839	0.85432677	0.84295441	0.85043303
$D^{*q}_{2}$ *AWP* q-red	0.86306276	0.85799349	0.84868427	0.85289041
*L*_2_	0.86125521	0.85265296	0.84487856	0.84694314
*D*_2_	0.85971734	0.85283644	0.84325115	0.84899721
KL	**0.86990625**	0.85621086	0.84559916	0.85108524
Simm, KL	0.86827273	0.85433859	0.84321338	0.85010800
**3000 reads**				
$D^{*}_{2}$	0.86131992	0.83027426	0.79355066	0.81057286
$D^{*q}_{2}$ *AQP*	0.86134064	**0.86519721**	**0.86235323**	**0.85792626**
$D^{*q}_{2}$ *AQP* q-red	0.86128705	0.85978356	0.85252267	0.85262847
$D^{*q}_{2}$ *AWP*	0.86477422	0.85334750	0.84374378	0.84947286
$D^{*q}_{2}$ *AWP* q-red	0.86477422	0.85637033	0.84850933	0.85162186
*L*_2_	0.86370337	0.85297951	0.84525794	0.84901375
*D*_2_	0.86242736	0.85271505	0.84384526	0.84832590
KL	**0.86934393**	0.85488377	0.84531374	0.85014251
Simm, KL	0.86580244	0.85353783	0.84308462	0.84878825
$D^{*}_{2}$	0.86179886	0.83217374	0.79345107	0.80917623
$D^{*q}_{2}$ *AQP*	0.86166330	**0.86412834**	**0.86385592**	**0.86064860**
$D^{*q}_{2}$ *AQP* q-red	0.86166519	0.85559541	0.85133437	0.85345570
$D^{*q}_{2}$ *AWP*	0.86317541	0.85224352	0.84168072	0.84837070
$D^{*q}_{2}$ *AWP* q-red	0.86317541	0.85543020	0.84770910	0.85121979
*L*_2_	0.86262435	0.85243814	0.84436053	0.84898583
*D*_2_	0.86122271	0.85167640	0.84308556	0.84801094
KL	**0.86792997**	0.85473650	0.84431637	0.84985690
Simm, KL	0.86488656	0.85297623	0.84262083	0.84815285

Best results are in bold.

The future generation sequencing technologies will produce long reads with a large number of erroneous bases. To this end we study how read length affects these measures. Since the length of sequences under investigation is limited we keep the read length under 400 bases. In Table 4 we report some experiments for the setup with 4 clusters and *k*=3, while varying the error rate and read length. If we compare these results with Table 2, where the read length is 200, we can observe a similar behavior. As the error rate increases the improvement with respect to the other measures remains evident, in particular the difference in terms of recall of $D^{*q}_{2}$ with the expectations *AQP* grows with the length of reads when compared with *KL* (up to 9%), and it remains constant when compared with $D^{*}_{2}$. With the current tendency of the future sequencing technologies to produce longer reads this behavior is desirable. These performance are confirmed also for other setups with larger *k* and higher number of clusters (data not shown).

**Table 4:** **Recall rates for clustering of mRNA simulated reads(10000 reads,** ***k*** **=3, 4 clusters) for different measures, error rates and read length**

	**read length = 300**					**read length = 400**			
	**(a)**					**(b)**			
**Distance**	**No errors**	**3** ***%***	**5** ***%***	**10** ***%***		**No Errors**	**3** ***%***	**5** ***%***	**10** ***%***
	**4 clusters**					**4 clusters**			
$D^{*}_{2}$	**0,680**	0,667	0,658	0,625		**0,713**	0,700	0,697	0,672
$D^{*q}_{2}$ *AQP*	**0,680**	**0,672**	**0,673**	**0,650**		**0,713**	**0,712**	0,710	0,693
$D^{*q}_{2}$ *AQP* q-red	**0,680**	0,671	**0,673**	**0,650**		**0,713**	0,711	**0,711**	**0,694**
$D^{*q}_{2}$ *AWP*	0,616	0,610	0,608	0,601		0,643	0,636	0,632	0,623
$D^{*q}_{2}$ *AWP* q-red	0,616	0,610	0,607	0,602		0,643	0,635	0,631	0,622
*L*_2_	0,610	0,600	0,602	0,581		0,638	0,630	0,624	0,614
KL	0,617	0,604	0,601	0,577		0,649	0,632	0,628	0,618
Symm, KL	0,613	0,603	0,599	0,576		0,647	0,632	0,627	0,616
*D*_2_	0,601	0,593	0,588	0,575		0,626	0,618	0,615	0,604

Best results are in bold.

### Boosting assembly

Assembly is one of the most challenging computational problems in the field of NGS data. It is a very time consuming process with highly variable outcomes for different datasets [34]. Currently large datasets can only be assembled on high performance computing systems with considerable CPU and memory resources. Clustering has been used as preprocessing, prior to assembly, to improve memory requirements as well as the quality of the assembled contigs [21,22]. Here we test if the quality of assembly of real read data can be improved with clustering. For the assembly component we use Velvet [35], one of the most popular assembly tool for NGS data. We study two genomes: *Helicobacter Pylori* and *Zymomonas Mobilis*. We download the reads datasets *SRR023794* and *SRR017901*, of about 117 and 23.5 MBases respectively, corresponding to 10 × coverage. We apply the clustering algorithms, with *k*=3, and divide the datasets of reads in two and three clusters. Then we produce an assembly, as a set of contigs, for each cluster using Velvet and we merged the generated contigs. In order to evaluate the clustering quality, we compare this merged set with the assembly, without clustering, using of the whole set of reads. Commonly used metrics such as number of contigs, *N*50 and percentage of mapped contigs are presented in Tables 5 and 6. When merging contigs from different clusters, some contig might be very similar or they can cover the same region of the genome, this can artificially increase these values. Thus we compute also a less biased measure that is the percentage of the genome that is covered by the contigs (last column).

**Table 5:** **Comparison of assembly with and without clustering preprocess (** ***k*** **=3, 2 clusters)**

**Distance**	**Mapped contigs**	**N50**	**Number of contigs**	**Genome coverage**
No Clustering	93.55%	112	22823	0,828
$D^{*q}_{2}$ *AQP* q-red	94.13%	**141**	**29421**	**0,920**
$D^{*}_{2}$	93.97%	138	28701	0,914
*L*_2_	94.24%	135	28297	0,904
KL	94.19%	135	28171	0,903
Symm, KL	94.27%	134	27999	0,902
*D*_2_	**94.33%**	134	28019	0,903

The assembly with Velvet is evaluated in terms of mapped contigs, N50, number of contigs and genome coverage. The dataset used is SRR017901 (23.5M bases, 10x coverage) that contains reads of *Zymomonas mobilis*. Best results are in bold.

**Table 6:** **Comparison of assembly with and without clustering preprocess (** ***k*** **=3, 3 clusters)**

**Distance**	**Mapped contigs**	**N50**	**Number of contigs**	**Genome coverage**
No Clustering	96.97%	122	16724	0.729
$D^{*q}_{2}$ *AQP* q-red	**98.49%**	175	**41086**	**0.994**
$D^{*}_{2}$	98.38%	174	40156	**0.994**
*L*_2_	98.16%	175	36798	0.986
KL	98.28%	178	37717	0.990
Simm, KL	98.30%	182	37217	0.990
*D*_2_	98.22%	**186**	34866	0.987

The assembly with Velvet is evaluated in terms of mapped contigs, N50, number of contigs and genome coverage. The dataset used is SRR023794 (117MBases) that contains reads of *Helicobacter Pylori*. Best results are in bold.

In this set of experiments the introduction of clustering as a preprocessing step increases the number of contigs and the N50. More relevant is the fact that the genome coverage is incremented by 10% with respect to the assembly without clustering. The relative performance between the distance measures is very similar to the case with simulated data. In fact $D^{*q}_{2}$ with expectation *AQP* and quality redistribution is again the best performing. More experiments should be conducted in order to prove that assembly can benefit from the clustering preprocessing. However this first preliminary tests show that, at least for some configuration, a 10% improvement on the genome coverage can be obtained. The time required to performed the above experiments are in general less than a minute on a modern laptop with an Intel i7 and 8Gb of ram. The introduction of quality values typically increases the running time by 4% compared to standard alignment-free methods.

### Clustering metagenomic reads

Another application, where the use of clustering techniques might be of help, is the classification of metagenomic reads. Modern sequencing machines are capable of sequencing several genomes at the same time, more precisely the input can be a microbiome community composed of thousands of different organisms. If the reference genomes are not available, or we don’t know all the organisms being sequenced, clustering techniques can be used to group together reads with the same word distribution that presumably come from the same genome. To test our quality based measure on this challenging task we devise a simple preliminary test. We consider the reads of the following four different organisms: *Helicobacter pylori* (*SRR023794*), *Zymomonas mobilis* (*SRR017901*), *E.coli* (*FXAWNEV04*) and *Legionella pneumophila* (*ERR164429*). These datasets contain reads of length between 150 to 350 bases. We create a single mixture of reads by sampling the same number of reads from each organisms. Then we tested how well clustering techniques can recover the original taxonomy of each genome in this artificial dataset. In Table 7 we report the recall rates for different alignment-free measures. Surprisingly, without knowing any reference genome, we can classify correctly about 80*%* of reads. Again quality based methods have a small advantage over traditional alignment-free techniques. This is just a preliminary test, however we believe that the classification of metagenomic reads with alignment-free methods deserved to be further investigated.

**Table 7:** **Metagenomic reads classification of** ***Helicobacter pylori*** **(** ***SRR023794*** **),** ***Zymomonas mobilis*** **(** ***SRR017901*** **),** ***E.coli*** **(** ***FXAWNEV04*** **) and** ***Legionella pneumophila*** **(** ***ERR164429*** **)**

**Distance**	**4 cluster**	**3 cluster**
$D^{*}_{2}$	0.79782297	0.79129356
$D^{*q}_{2}$ *AQP* q-red	0.79775189	0.76920676
$D^{*q}_{2}$ *AWP* q-red	**0.80050234**	**0.82603989**
*L*_2_	0.64335292	0.73455525
KL	0.78663484	0.80525234
Simm, KL	0.77196713	0.79216786
*D*_2_	0.73917085	0.77062424

The recall rates for different measures with *k* = 4 and 3 and 4 clusters. Best results are in bold.

## Conclusions

The comparison of reads with quality values is essentials in many genome projects. Moreover, the importance of quality values will increase in the near future with the advent of future sequencing technologies, that promise to produce long reads, but with up to 15% error rates. In this paper we presented a family of alignment-free measures, called *D*^*q*^-type, that incorporate quality value information and *k*-mers counts for the comparison of reads data. A set of experiments on simulated and real reads data confirms that the new measures are superior to other classical alignment-free statistics, especially when erroneous reads are considered. If quality information are used, $D^{*q}_{2}$ outperforms all other methods and the advantage grows with the error rate and with the length of reads. This confirms that the use of quality values can improve clustering accuracy.

Furthermore, preliminary experiments on real reads data show that the quality of assembly can be improved by using clustering as preprocessing. Also metagenomic reads classification can be addressed with these statistics, especially when the reference genomes are unknown. All these measures are implemented in a software called QCluster. As a future work we plan investigate other applications like genome diversity estimation and meta-genome assembly in which the impact of reads clustering might be substantial.

## Endnote

^a^ a preliminary version of this work as been presented at WABI 2014 [36].
